# Association of *interleukin 4* and *MTHFR* gene polymorphisms with distal symmetrical polyneuropathy in young diabetics

**DOI:** 10.1055/s-0044-1793931

**Published:** 2024-12-15

**Authors:** Raquel Garcia Rocco da Silva, Marcelo A. Costa Lima, Claudia de Melo Moura, Jorge Luiz Luescher, Ludmila Nascimento Rodrigues Campos, Daniel de Souza e Silva, Márcia Gonçalves Ribeiro

**Affiliations:** 1 Universidade Federal do Rio de Janeiro, Instituto de Puericultura e Pediatria Martagão Gesteira, Rio de Janeiro RJ, Brazil.; 2 Instituto Federal do Rio de Janeiro, Departamento de Saúde, Rio de Janeiro RJ, Brazil.; 3 Universidade do Estado do Rio de Janeiro, Instituto de Biologia Roberto Alcântara Gomes, Laboratório de Genética Molecular Humana, Rio de Janeiro RJ, Brazil.; 4 Pontífica Universidade Católica do Rio de Janeiro, Departamento de Medicina e Saúde, Rio de Janeiro RJ, Brazil.

**Keywords:** Diabetic Neuropathies, Child, Adolescent, Polymorphism, Genetic, Interleukin-4, Methylenetetrahydrofolate Reductase (NADPH2), Neuropatias Diabéticas, Criança, Adolescente, Polimorfismo Genético, Interleucina-4, Metilenotetra-hidrofolato Redutase (NADPH2)

## Abstract

**Background** It is believed that genetic factors play a role in the development and severity of neural injury among people with distal symmetrical polyneuropathy (DSP), because some genes are involved in specific biological pathways, acting in different ways in the pathogenic process.

**Objective** To identify potential associations involving the *5,10-methylenetetrahydrofolate reductase* ( *MTHFR* C677T) and *interleukin 4* ( *IL-4* intron 3 variable number of tandem repeats [I3VNTR]) gene polymorphisms and DSP in the studied sample.

**Methods** In total, 70 children and adolescents with type-1 diabetes underwent a nerve conduction studie (NCS) of the sural nerve. Saliva samples were collected for DNA extraction and genotyping of the *MTHFR* C677T and *IL-4* I3VNTR polymorphisms.

**Results** The prevalence of DSP was 15.71%. The participants with DSP presented higher mean levels of glycated hemoglobin, triglycerides, total cholesterol, and low-density lipoprotein (LDL) ( *p* > 0.05). The NCS amplitudes were lower in individuals with DSP ( *p* = 0.00). The mean conduction velocity was lower in people with the *A1A1* genotype ( *p* = 0.02). Maternal and paternal history of diabetes in great-grandparents were associated with DSP ( *p* = 0.04 and 0.02, respectively). Glycated hemoglobin and impaired Achilles reflex were associated with the *MTHFR* *CC* genotype ( *p* = 0.04 and 0.05 respectively) and high-density lipoprotein (HDL) cholesterol was associated with the *MTHFR* *CT* genotype ( *p* = 0.05). We found no association between the polymorphisms investigated and DSP.

**Conclusion** In the present study, we found no association involving the *MTHFR* C677T and *IL-4* I3VNTR polymorphisms and DSP. However, the study provides other associations and suggests possible implications for these findings.

## Resumo

**Antecedentes** Acredita-se que fatores genéticos desempenhem um papel no desenvolvimento e na gravidade da lesão neural em pessoas com polineuropatia simétrica distal (PSD), pois alguns genes estão envolvidos em vias biológicas específicas, e atuam de diferentes formas no processo patogênico.

**Objetivo** Identificar as possíveis associações entre os polimorfismos dos genes *5,10-metileno tetrahidrofolato redutase* ( *MTHFR* C677T) e *interleucina 4* ( *IL-4* íntron 3 número variável de repetições em tandem, *intron 3 variable number of tandem repeats* [I3VNTR], em inglês) com a PSD na amostra estudada.

**Métodos** Ao todo, 70 crianças e adolescentes com diabetes tipo 1 foram submetidos ao estudo de condução neural (ECN) do nervo sural. Amostras de saliva foram coletadas para extração de DNA e genotipagem dos polimorfismos *MTHFR* C677T e *IL-4* I3VNTR.

**Resultados** A prevalência de PSD foi de 15,71%. Os participantes com PSD apresentaram níveis médios maiores de hemoglobina glicada, triglicerídeos, colesterol total, e colesterol de lipoproteínas de baixa densidade ( *low-density lipoprotein* , LDL, em inglês) ( *p* > 0,05). As amplitudes do ECN foram menores nos indivíduos com PSD ( *p* = 0,00). A média da velocidade do ECN foi menor nas pessoas com genótipo *A1A1* ( *p* = 0,02). Histórico materno e paterno de diabetes nos bisavós esteve associado à PSD ( *p* = 0,04 e 0,02, respectivamente). Não encontramos associação entre os genótipos investigados e a PSD. A hemoglobina glicada e a diminuição do reflexo aquileu foram associados ao genótipo *MTHFR* *CC* ( *p* = 0,04 e 0,05, respectivamente), e o colesterol de lipoproteínas de alta densidade ( *high-density lipoprotein* , HDL, em inglês) foi associado ao genótipo *MTHFR* *CT* ( *p* = 0,05).

**Conclusão** Neste estudo, não encontramos associação entre o polimorfismo C677T do gene *MTHFR* e o polimorfismo I3VNTR do gene *IL-4* . No entanto, o estudo fornece outras associações e sugere possíveis implicações para esses achados.

## INTRODUCTION

The clinical manifestations of type-1 diabetes mellitus (T1DM) are polymorphous and vary considerably among individuals. The microvascular repercussions are based on diabetic triopathy – retinopathy, nephropathy, and neuropathy – and constitute an important health problem, resulting in morbidity and mortality and significantly worsening the quality of life due to disability and reduced survival. 1

Neuropathic involvement is usually early and encompasses a wide spectrum of abnormalities, affecting various types of sensory, motor, and autonomic fibers, occurring both in T1DM and type-2 diabetes mellitus (T2DM), as well as in acquired forms of diabetes, with distal symmetrical polyneuropathy (DSP) being the most common and known form of neuropathy. 2

The prevalence and incidence of DSP in individuals with T1DM vary considerably, but the estimates are that, after 20 years of disease duration, 20% of patients develop DSP. 3

Despite the fact that glycemic control and the duration of diabetes are considered important factors for the development of DSP, it has been observed 4 that the speed of progression and involvement of the nervous tissue present great individual differences. There is evidence that genetic predisposition plays a role in the susceptibility to nerve damage. With the introduction of molecular biology analysis in different areas, some polymorphisms have been identified, which may be related to the complications of T1DM. 5 6 7 8

The genes responsible for favoring an inflammatory response are important candidates associated with neural damage. 5 Interleukin-4 (IL-4) is a cytokine secreted by type-2 T cells, eosinophils, and macrophages, and it plays an important role in the formation of the inflammatory response of endothelial cells. Several polymorphisms of the *IL-4* gene have been described, and an intronic variable number of tandem repeats (VNTR) is associated with inflammatory diseases and a deficiency in the regulation and production of IL-4. 6 7

Hyperhomocysteine is also linked to impaired nerve function due to oxidative stress. *Methylenetetrahydrofolate reductase* ( *MTHFR* ) catalyzes the transformation of homocysteine into methionine through the remethylation pathway; however, a decrease in *MTHFR* activity due to mutation increases the chances of hyperhomocysteinemia. 8 9 10 11

Therefore, the present study aimed to identify a potential association involving the C677T polymorphisms of the *MTHFR* gene and the intron 3 (I3) VNTR polymorphisms of the *IL-4* gene and distal symmetrical polyneuropathy.

## METHODS

### Definition of the case and universe of the study

The present is a cross-sectional observational study of 70 children and adolescents with T1DM. The inclusion criteria were: literate children and adolescents of both sexes aged ≤ 18 years; T1DM diagnosis for ≥ 5 years; ability to understand the exam; and passing the compliance test, which identifies the tendency to agree. The exclusion criteria were: presence of neuropathies caused by other etiologies or other diseases of the central and peripheral nervous system, use of metformin, 12 vitamin B12 deficiency, 12 and hypothyroidism, all recorded in the medical records; and asymmetric sensory and motor deficits. 2 A content-structured questionnaire was applied.

### Neurological assessment

The clinical evaluation consisted of muscle strength (quadriceps femoris and tibialis anterior), reflex (triceps surae and patellar), and sensitivity (painful sensitivity in the second finger and in the hallux, sensitivity to touch in the hallux, perception of vibration in the hallux, hallux joint position, protective sensitivity, and thermal sensitivity of the dorsum of the foot).

### Samples, DNA extraction, and molecular genotyping

All participants provided salivary material to obtain genomic obtained by swishing 3 mL of sucrose solution at 3%, and stored at -20°C.

The *MTHFR* C677T polymorphism was analyzed by polymerase chain reaction-restriction fragment length polymorphism (PCR-RFLP). The amplification conditions consisted of 5 minutes at 94°C, followed by 35 cycles of 30 seconds at 94°C, 30 seconds at 61°C, and 30 seconds at 72°C, with a final elongation step of 5 minutes at 72°C. The PCR was performed in a 25-µL reaction containing 100 ng of DNA, 2.5 µL of 10X PCR buffer, 200 µM of deoxynucleoside triphosphates (dNTPs), 10 picomolars (pM) of each primer ( *5′-TGAAGGAGAAGGTGTCTGCGGGA-3′* and *5′-AGGACGGTGCGGTGAGAGTG-3′* ), and 1 unit (U) of Taq DNA polymerase. After amplification, the PCR product was digested in 15 µL of reaction solution containing 10 µL of PCR product, 1.5 µL of 10X buffer and 2 U HinfI at 37°C, and the products were resolved on 3% agarose gels and visualized under ultraviolet (UV) light after ethidium bromide staining. The wild-type *C* allele is a single 198-base pair (bp) fragment, while the mutant *T* allele is cleaved into 175- and 23-bp fragments.

The *IL-4* I3VNTR polymorphism was analyzed by PCR-simple sequence length polymorphism (PCR-SSLP). Amplification was performed in a 25-μL reaction containing 3 µL of DNA, 10 μL of PCR reaction buffer, 2 μL of each primer ( *5′-GTAAATAGGCTGAAAGGGGGAAA-3* and *5′-CATCTTTTCCTCCCCTGTATCTT-3′* ), and 1 U of Taq DNA polymerase. The amplification conditions consisted of 2 minutes at 95°C, followed by 35 cycles of 1 minute at 95°C, 1 minute at 56°C, and 30 seconds at 72°C, with a final elongation step of 5 minutes at 72°C. The PCR products were electrophoresed on 2.5% agarose gels and visualized under UV light after ethidium bromide staining. The wild-type allele contains 3 VNTRs (3R – named *A1* ), and it appears as a 342-bp fragment, while the rare allele containing 2 VNTRs (2R – named *A2* ) is visualized as a 272-bp fragment. The genotypes are represented by *A1A1* (homozygous wild-type), *A1A2* (heterozygous), and *A2A2* (homozygous variant).

### Parameters and evaluation of nerve conduction

After the children and adolescents were evaluated at the diabetes clinic, they were invited to attend Hospital Universitário Pedro Ernesto for nerve conduction studies (NCSs) using the Counterpoint instrument (Medtronic, Minneapolis, MN, United States). Surface electrodes were positioned on the lateral edge of the foot, close to the fifth toe, with the stimulus site posterior to the lateral malleolus, and the cathode located ∼ 100 mm proximal to the recording electrode. The evaluation was bilateral, that is, it involved the right foot and left foot. A normal reference record for the electrophysiological study was considered: action potential amplitude (µV) > 6 and conduction velocity (m/s) > 39. 13

The diagnosis of DSP was established through the presence of NCS abnormality. 14

### Statistical analysis

The prevalence of DSP and its 95% confidence interval (95%CI; *p* = 0.05) were estimated, as well as the odds ratio (OR) for the investigated variables. Analysis of variance (ANOVA) was used to compare the means. Data were analyzed using the Epi-Info statistical package (Centers for Disease Control and Prevention, Atlanta, GA, United States), version 7.2. Statistical significance was assessed using the Chi-squared (χ ^2^ ) test for proportions and Student's *t* - or Kruskal–Wallis tests for means. A logistic regression model was fitted to identify the independent factors associated with DSP.

The project of the present study was approved by the Research Ethics Committee of Instituto de Puericultura e Pediatria Martagão Gesteira/Universidade Federal do Rio de Janeiro (IPPMG/UFRJ) under number 1.910.920 (CAAE 60302816.7.0000.5264). Written informed consent was obtained from all participants.

## RESULTS

**Figure 1:**
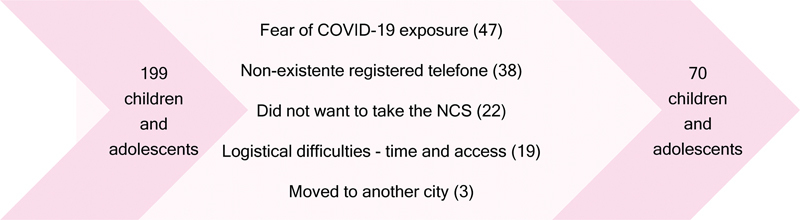
Children and adolescents taking part in the study.

In total, 70 children and/or adolescents underwent NCS of the sural nerve ( Figure 1 ). The prevalence of DSP was of 15.71% (27.27% among girls and 72.73% among boys), and the statistical association with gender was at a significance level of 10% ( *p* = 0.07). Table 1 shows the mean amplitudes and velocities of the NCS.

**Table 1:** Mean of the parameters evaluated in the NCSs of the sural nerve and their associations with DSP

Parameter	With DSP: mean ± SD	Without DSP: mean ± SD	*p* -value
Sural nerve – right amplitude (mV)	5.52 ± 2.99	16.81 ± 5.80	**0.00**
Sural nerve – left amplitude (mV)	6.26 ± 3.23	16.40 ± 6.17	**0.00**
Sural nerve – right conduction velocity (m/s)	52.44 ± 8.06	50.79 ± 4.81	0.40
Sural nerve – left conduction velocity (m/s)	51.44 ± 5.41	51.26 ± 5.27	0.92

Abbreviations: DSP, distal symmetrical polyneuropathy; NCSs, nerve conduction studies; SD, standard deviation.

In Table 2 , we the observe clinical and laboratory variables and their associations with DSP, before and after stratification by sex.

**Table 2:** Clinical and laboratory variables and their associations with DSP

Clinical and laboratory variables	Overall sample – with DSP; without DSP: mean ± SD	*p* -value	Girls* – with DSP; without DSP: mean ± SD	*p* -value*	Boys* – with DSP; without DSP: mean ± SD	*p* -value*
Age (years)	15.27 ± 2.61; 15.57 ± 2.35	0.70	17.33 ± 1.52; 15.25 ± 1.93	0.08	14.50 ± 2.56; 15.92 ± 2.73	0.19
Time since diabetes diagnosis (years)	11.45 ± 2.33; 11.46 ± 2.68	0.98	12.33 ± 2.08; 10.96 ± 2.34	0.33	11.12 ± 2.47; 12.00 ± 2.95	0.44
Glycated hemoglobin** (%)	9.58 ± 3.40; 7.95 ± 0.68	**0.00**	8.54 ± 1.12; 8.18 ± 0.60	0.38	10.03 ± 4.01; 7.69 ± 0.68	**0.00**
Weight (Kg)	62.02 ± 17.83; 59.43 ± 11.86	0.54	69.00 ± 14.42; 58.59 ± 10.60	0.12	59.41 ± 19.14; 60.34 ± 13.21	0.83
Height (cm)	1.65 ± 0.15; 1.64 ± 0.10	0.72	1.71 ± 0.04; 1.60 ± 0.06	**0.01**	1.62 ± 0.16; 1.67 ± 0.12	0.41
Body mass index (Kg/m ^2^ )	22.26 ± 3.71; 21.95 ± 2.84	0.75	23.45;4.38; 22.67 ± 3.11	0.69	21.82 ± 3.66; 21.19 ± 2.34	0.55
Systolic blood pressure (mmHg)	105.45 ± 14.39; 105.42 ± 9.89	0.99	96.66 ± 5.77; 102.62 ± 7.09	0.17	108.75 ± 15.52; 108.32 ± 11.56	0.93
Diastolic blood pressure (mmHg)	65.45 ± 6.87; 66.78 ± 8.04	0.60	63.33 ± 5.77; 65.24 ± 6.62	0.63	66.25 ± 7.44; 68.39 ± 9.13	0.54
Microalbuminuria (g/dL)	10.96 ± 8.09 7.21 ± 6.73	0.12	13.70 ± 10.55; 8.83 ± 7.88	0.33	9.79 ± 7.45; 5.22 ± 4.35	**0.05**
HDL cholesterol (mg/dL)	60.09 ± 15.74; 59.39 ± 15.68	0.89	56.66 ± 19.50; 64.03 ± 14.76	0.42	61.37 ± 15.44; 54.42 ± 15.35	0.26
LDL cholesterol(mg/dL)	108.27 ± 47.70 84.55 ± 25.77	**0.01**	183.33 ± 91.62; 92.96 ± 25.47	**0.03**	97.00 ± 17.88; 75.53 ± 23.28	**0.01**
Triglycerides (mg/dL)	94.72 ± 58.08; 66.82 ± 35.89	**0.03**	106.66 ± 74.23 74.70 ± 38.08	0.21	90.25 ± 56.22; 58.39 ± 31.92	**0.04**
Total cholesterol (mg/dL)	187.18 ± 62.28; 156.31 ± 35.71	**0.02**	216.33 ± 126.15; 168.83 ± 35.91	0.10	176.25 ± 22.23; 142.89 ± 30.78	**0.00**
VLDL cholesterol (mg/dL)	22.60 ± 11.99; 14.90 ± 9.11	0.10	38.00 ± 0.00 17.18 ± 10.09	0.09	18.75 ± 9.63; 12.46 ± 7.53	0.10

Abbreviations: DSP, distal symmetrical polyneuropathy; HDL, high-density lipoprotein; LDL, low-density lipoprotein; SD, standard deviation; VLDL, very-low-density lipoprotein.

Notes: *Adjusted by sex. **The means of eight measurements for each patient were analyzed.

Regarding maternal history, we observed a 3.26 higher risk of developing DSP among children and adolescents who had great-grandparents with T1DM (95%CI = 1.12–9.45; *p* = 0.04). Paternal history of great-grandparents with T1DM was also associated with DSP (OR = 3.43; 95%CI = 1.22–9.60; *p* = 0.02).

**Figure 2:**
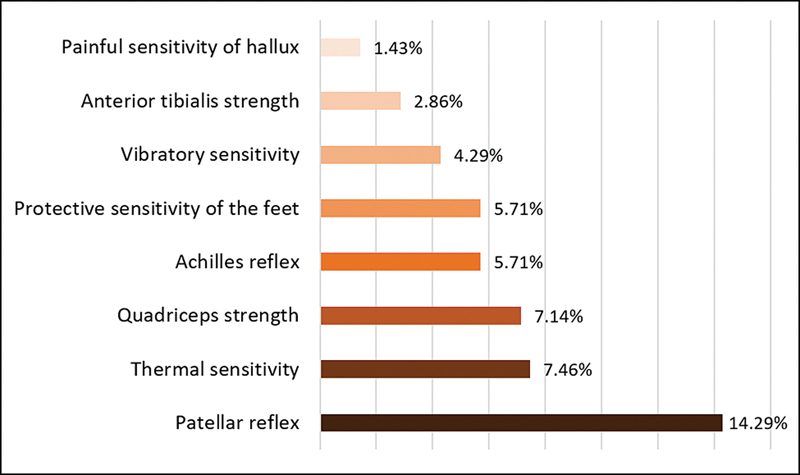
Frequency of alterations observed in the neurological evaluation.

The frequency of clinical signs evaluated is shown in Figure 2 . Patients with DSP had a 4.87 higher risk of presenting changes in thermal sensitivity than patients without DSP (95%CI = 1.85–12.80; *p* = 0.01). The associations were also present for changes in the Achilles reflex (OR = 6.18; 95%CI = 2.61–14.64; *p* = 0.00) and in the patellar reflex (OR = 5.00; 95%CI = 1.87–13.32; *p* = 0.00). Regarding the protective sensitivity of the feet, despite the 95% CI being associated between 95%, the *p* -value was higher than 0.05 (OR = 3.66; 95%CI = 1.15–11.61; *p* = 0.06).

**Figure 3:**
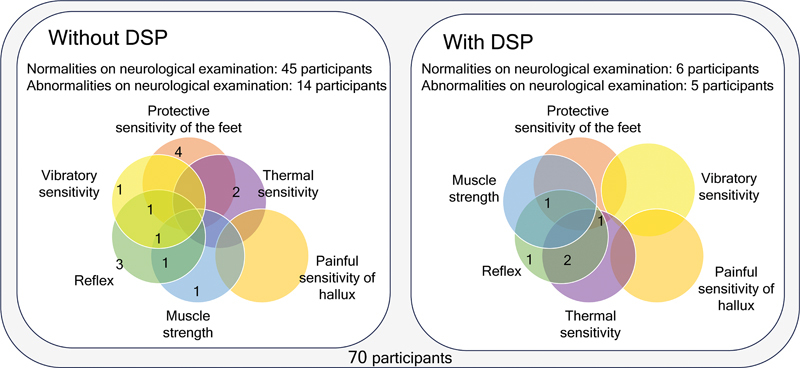
Note: Venn Diagram. Frequency of alterations observed in the neurological evaluation among participants with and without distal symmetrical polyneuropathy (DSP).

Figure 3 shows the frequency of alterations observed in the neurological evaluation of the participants with and without DSP.

The *IL-4* I3VNTR *A1A1* genotype was more frequent in the study sample (61.40%), followed by the *A1A2* (35.09%) and *A2A2* (3.51%) genotypes. The frequencies of the *A1* and *A2* alleles were of 69% and 31% respectively. The mean values for conduction velocity were lower in individuals with the *A1A1* genotype (50.01 m/s versus 53.20 m/s; *p* = 0.02).

The homozygous wild-type *MTHFR* ( *CC* ) genotype was the most prevalent in the population (55.88%), followed by the heterozygote ( *CT* ; 39.71%) and homozygous variants ( *TT* ; 4.41%). The *C* allele was present in 68% of the patients, and the *T* allele, in 32%. Table 3 shows the frequency of *IL-4* and *MTHFR* genotypes and alleles of patients with and without DSP; no statistical associations with DSP were observed.

**Table 3:** *IL-4* and *MTHFR* genotypes between patients with and without DSP

Polymorphisms		Individuals: %		*p* -value
		with DSP	without DSP	
*IL-4*	* A1A1*	15.00	85.00	0.42
	* A1A2*	19.05	80.95	0.31
	* A2A2*	14.29	85.71	0.49
Allele *A1*		16.39	83.61	0.38
Allele *A2*		17.86	82.14	0.34
*MTHFR*	* CC*	18.42	81.58	0.29
	* CT*	14.81	85.19	0.41
	* TT*	0.00	100.00	*
Allele *C*		16.92	83.08	0.20
Allele *T*		13.33	86.67	0.33

Abbreviations: *A1A1* , homozygous wild-type; *A1A2* , heterozygous; *A2A2* , homozygous variant; *CC* , homozygous wild-type individuals; *CT* , heterozygous individuals; DSP, distal symmetrical polyneuropathy; *IL-4,* interleukin 4; *MTHFR,* methylenetetrahydrofolate reductase; *TT* , homozygous individuals.

The investigated *IL-4* and *MTHFR* genotypes were not associated with any clinical sign. The change in the Achilles reflex was associated with the *MTHFR* *CC* polymorphism ( *p* = 0.04) ( Table 4 ).

**Table 4:** Clinical variables investigated and their associations with *IL-4* and *MTHFR* genotypes

Clinical variables*	*IL-4* : OR ( *p* -value)			*MTHFR* : OR ( *p* -value)		
	*A1A1*	*A1A2*	*A2A2*	*CC*	*CT*	*TT*
Quadriceps femoris strength	0.68 (0.23)	2.16 (0.09)	*	1.48 (0.15)	0.48 (0.20)	*
Anterior tibialis strenght	1.78 (0.16)	*	*	0.89 (0.44)	1.26 (0.39)	*
Patelar reflex	1.05 (0.43)	1.41 (0.23)	*	1.08 (0.39)	0.72 (0.26)	2.90 (0.21)
Achillesreflex	1.33 (0.26)	0.82 (0.44)	*	**1.88 (0.04)**	*	*
Painful sensitivity of the second finger	*	*	*	*	*	*
Painful sensation of the great toe	1.76 (0.28)	*	*	*	2.57 (0.19)	*
Hallux touch sensitivity	*	*	*	*	*	*
Hallux vibration sensitivity	1.17 (0.39)	1.11 (0.43)	*	1.20 (0.37)	0.83 (0.43)	*
Hallux joint perception	*	*	*	*	*	*
Thermal sensitivity	1.44 (0.16)	0.72 (0.14)	*	1.48 (0.15)	0.48 (0.20)	*
Protective foot sensitivity	1.33 (0.26)	0.82 (0.44)	*	0.88 (0.41)	1.28 (0.34)	*

Abbreviations: *A1A1* , homozygous wild-type; *A1A2* , heterozygous; *A2A2* , homozygous variant; *CC* , homozygous wild-type individuals; *CT* , heterozygous individuals; *IL-4,* interleukin 4; *MTHFR,* methylenetetrahydrofolate reductase; OR, odds ratio; *TT* , homozygous individuals.

Note: *Corresponds to changes in strength, reflexes and sensitivity.

The means values for glycated hemoglobin were higher in patients with the *MTHFR* *CC* genotype (8.58% versus 7.78%; *p* = 0.05). In the *MTHFR* CT genotype, HDL cholesterol showed lower mean values (55.15 mg/dL versus 62.75 mg/dL; *p* = 0.05).

## DISCUSSION

To the best of our knowledge, no studies have been conducted with Brazilian children and adolescents with T1DM on the genetic investigation of DSP with the polymorphisms presented.

Knowledge of the prevalence of DSP is extremely important, because DSP is a multifactorial disease related to genetic, environmental and ethnic risks. Therefore, its frequency corroborates actions related to education, promotion, and administration in health. 15 In the present study, we were able to estimate the prevalence of DSP in a population of children and adolescents who are patients at an outpatient clinic at an university hospital and are routinely followed, minimizing possible information biases. We observed that 15.71% of them had DSP, and we did not find studies in Brazil that aimed to estimate this frequency in children and adolescents with T1DM.

In the literature, we observed a wide variation in the frequency of DSP (7% to 90%), which may be related to the diagnostic criteria and instruments assigned in each study. 16

We use sensory NCS of bilateral sural nerves to diagnose DSP in our patients, independently of the clinical findings. 14 There is no consensus regarding which peripheral nerve offers greater sensitivity and specificity to detect DSP. 2 Some studies propose an evaluation of motor neves 14 and others recommend the sensory nerves. 13 14 In the present study, we observed that the amplitudes of the NSC were reduced and associated with PSD and, as we only assessed the sural nerve, it was not possible to evaluate these relationships. Balci et al. (2005) 13 observed that the sural nerve is a reliable parameter for the early identification of PSD. The predominant abnormality of the NCS is the loss of axons, which, electrophysiologically, means a reduction in the amplitudes, not the speeds, of nerve conduction and, therefore, the changes observed may not be the best strategy to monitor the progression of nerve damage. 14

Regarding the natural history of DSP, we observed asynchrony in relation to the frequencies of nerve damage in the present study as commonly indicated in the literature. 14 Longer axons are more susceptible to cell injury, 17 because PSD classically presents with length-dependent symptoms and signs, but we observed more frequent alterations in more proximal components than in distal components: quadriceps femoris (7.14%) versus tibialis anterior (2.86%) and patellar reflex (14.29%) versus Achilles reflex (2.86%). Nevertheless, among girls, height was statistically associated with DSP ( *p* = 0.01). In addition, small sensory fibers are often the first to be affected; nonetheless, we observed that alterations in the osteotendinous reflex (14.29%) were more frequent than alterations in thermal (7.46%) and pain (1.43%) sensitivity. This result may be due to the fact that the study sample was composed of children and adolescents; therefore, we were able to find initial lesions or isolated small fiber neuropathy. Thus, we only use the NCS as a diagnostic criterion to avoid including children and/or adolescents with small fiber lesions without using a gold-standard instrument (skin biopsy).

With regard to the lipid profile, it has been postulated that there is a linear relationship between lipid concentrations and microvascular changes, and that reaching lower serum concentrations brings clinical benefits to the population. 18 We did not classify the lipid profile in the study sample, but we observed that the serum levels of triglycerides, LDL and total cholesterol were higher in patients with DSP ( *p* = 0.01; 0.03; and 0.02, respectively). Dyslipidemia is multifactorial, but it is related to poor glycemic control, insulin resistance, and genetic susceptibility. 19 Glycated hemoglobin was associated with DSP in boys, and their relationship has already been disseminated in large studies on T1DM. 3 20 Hyperglycemia induces two pathways responsible for neural damage: the metabolic and ischemic pathways, which are closely related. The activation of these pathways triggers successive inflammatory and ischemic processes that culminate in impaired nerve conduction, neurovascular dysfunction, apoptosis, and sensory deficits. 21

Microvascular complications have been found to be more prevalent in adolescent girls than in boys; 22 however in the sample of the present study, although there was no statistical significance, the association was lower than 10% ( *p* = 0.07). Although we did not observe an association between DSP and gender, we observed a statistical association between the male sex and the amplitude of the ECN, with boys presenting lower mean values ( *p* = 0.01). After the stratification by sex, microalbuminuria was associated with DSP among boys ( *p* = 0.05).

Evidence indicates that genetic susceptibility influences microvascular dysfunction, 15 and we observed both maternal and paternal diabetes history in great-grandparents ( *p* = 0.04 and 0.02 respectively). We did not find any studies on diabetes history beyond the paternal and maternal relationship.

Autoimmunity has also been proposed as a pathogenic mechanism for DSP. 23 Interleukin-4 plays a key role in autoimmunity and acts as an anti-inflammatory; 7 it is a type of chemokine and cytokine, and it acts as a mediator of inflammatory or proinflammatory functions. 6 We did not observe statistical associations involving the IL-4 I3VNTR polymorphisms and DSP, but we observed lower mean values for conduction velocity in young with the A1A1 Q23 genotype ( *p* = 0.02), suggesting a possible risk factor in this relationship. The *IL-4* gene regulates lipid and glucose metabolism, 24 25 and the *A1* allele, compared with the *A2* allele, has a lesser deleterious effect. 26 However, we did not observe associations regarding *IL-4* genotypes and risk factors related to DSP.

The association of these polymorphisms with DSP also strongly correlated them with cardiovascular diseases, which are more frequent in adults. 7 This makes us think that the fact that our sample consisted of children and adolescents perhaps points to the fact that the deleterious effects are related to the time of exposure, that is, to age. We did not find any studies on children and adolescents with T1DM. Inflammation is a major contributor to neuronal injury.

The *MTHFR* C677T polymorphism produces a thermolabile variant that presents a reduced activity and increases homocysteine levels. Heterozygous individuals ( *CT* ) have ∼ 60% of the enzymatic activity compared with wild-type homozygotes ( *CC* ), while mutant homozygotes ( *TT* ) present only 30% of the enzymatic activity. 27 28 The *T* allele was frequent in 32% and the *C* allele in 68% of the studied sample.The frequency of the *T* allele differs among countries and ethnicities, but the highest value (65%) was observed in Amerindians. 29

We found studies that aimed to evaluate the association between the C677T polymorphism and DSP, and the results were divergent. 9 30 31 32 33 34 35 36 We observed that the population samples differed considerably among studies regarding sample size, nationality, lifecycle, diagnostic criteria, and type of diabetes. In addition, no study has analyzed children and adolescents. The association between the *MTHFR* C677T polymorphism and DSP was observed in 5 studies; 9 30 32 33 34 however,it was not observed in 3 studies. 31 35 36 In the present study, we found no statistically significant association with any of the genotypes analyzed, even after adjusting for possible confounding variables. Wild-type homozygous individuals ( *CC* ) present better enzymatic activity than heterozygotes ( *CT* ) and those with the homozygous variant ( *TT* ). However, the *CC* genotype was associated as a risk factor for alteration of the Achilles reflex ( *p* = 0.04), and the *CT* genotype was associated with glycated hemoglobin ( *p* = 0.05) and HDL cholesterol ( *p* = 0.05).

## Limitations and suggestions

Jaiswal et al. (2017) 16 suggested that the different tonicities among young people and adults with T1DM and their relationships with microvascular complications need to be better understood; however, we did not assess the phenotype and its possible relationships with DSP. Gene expression is also influenced by epigenetic mechanisms. 16 Despite having collected clinical and laboratory variables, we believe that there may be another mechanism that sheds light on a possible functional interference of the polymorphism.

Arruda et al. (1998) 37 conducted a study to determine the prevalence of the C677T mutation among different ethnic groups, and they observed that 10% of Caucasians, 1.45% of Blacks, and 1.2% of Brazilian indigenous individuals were carriers of the homozygous variant. The absence of participants with the homozygous variant, especially among those with DSP, made it impossible to analyze their associations. In the present study, the frequency of double mutants was of 7.04%, but we did not record ethnicity to consider this relationship. Heterozygous children and adolescents represented 39.71%, and those homozygous, 60.29%. In the study by Arruda et al., 37 the frequency of homozygosity was higher among Black and indigenous individuals (62.75% and 79.7%, respectively).

The prevalence of C677 polymorphism is related to ethnicity, and, in Brazil, its origin is extremely heterogeneous. 37 We believe that the lack of information on ethnicity may have generated a confounding factor, which could be perfectly controlled in the statistical analysis, making it impossible to assess the relationship between allele frequencies and polymorphisms, as well as their relationships with neural injury.

Another important point is that the genetic variant *MTHFR* C77T predisposes to reduced enzymatic activity (hyperhomocysteine) and lower levels of folate and of vitamins B6 and B12. 38 Therefore, the introduction of vitamins and minerals into the dietary routine can minimize the enzyme deficiency caused by the C677T polymorphism. 39 Because we did not assess the intake of vitamins and minerals, we could not analyze the confounding effects of this relationship. Measurement of homocysteine levels is a strategy to analyze its effect on neural injury. 40

In conclusion, we were able to characterize a sample of Brazilian children and adolescents with T1DM who were systematically monitored by a reference outpatient clinic at a university hospital, and we estimate the prevalence of DSP at 15.7%.

Although we did not observe an association between the C677T polymorphism of the *MTHFR* gene and the I3VNTR polymorphism of the *IL-4* gene, the present study provides other associations and suggests possible implications for the findings. We observed some associations with the *MTHFR* *CC* genotype (glycated hemoglobin and Achilles reflex impairment) and with the *MTHFR* *CT* genotype (HDL cholesterol).
